# Formative and Pilot Study for an Effectiveness-Implementation Hybrid Cluster Randomized Trial to Incorporate Oral Health Activities into Pediatric Well-Child Visits

**DOI:** 10.3390/dj8030101

**Published:** 2020-09-01

**Authors:** Suchitra Nelson, Mary Beth Slusar, Shelley Curtan, David Selvaraj, Andrew Hertz

**Affiliations:** 1 Department of Community Dentistry, Case Western Reserve University School of Dental Medicine, Cleveland, OH 44106-4905, USA; sgc36@case.edu (S.C.); dms256@case.edu (D.S.); 2 Department of Sociology, California State University Northridge, Northridge, CA 91330, USA; marybeth.slusar@csun.edu; 3 University Hospitals Cleveland Medical Center, Cleveland, OH 44106, USA; andrew.hertz@uhhospitals.org

**Keywords:** oral health, pediatric, qualitative research, pilot study, implementation

## Abstract

**Background**: Dental caries in pediatric patients are noted to have broad impacts on systemic health and well-being. Thus, utilizing an effectiveness-implementation hybrid I design, the Pediatric Providers Against Cavities in Children’s Teeth (PACT) trial is investigating multi-level interventions at the practice (incorporation of oral health in electronic medical record [EMR]) and provider levels (theory-based didactic and skills training to communicate oral health facts to parent/caregiver, give a prescription to see a dentist and a list of area dentists) to increase dental utilization among 3 to 6 year old Medicaid-enrolled children attending well-child visits (WCV). The formative and pilot work for the larger main trial are presented. **Methods**: Formative work—Focus groups with 26 participants (Community leaders, providers, parent/caregivers); and key informant interviews with practice leadership (n = 4). Topics discussed were: core oral health (OH) information to communicate at WCVs and study logistics. Transcripts were coded and analyzed using Atlas.ti; *Pilot study* was refined using the formative findings and was conducted at two pediatric practices to test the implementation of: the provider didactic and skills training curriculum; EMR incorporation of four OH questions; logistics of incorporating OH activities at a WCV; and parent/caregiver recruitment. **Results**: Formative work showed that providers and parent/caregivers required knowledge of dental caries, and a list of area Medicaid-accepting dentists. Providers and practice leadership advised on the logistics of incorporating oral health into WCVs. All groups suggested asking parent/caregivers their preferred method of contact and emphasizing importance of OH to motivate participation. Utilizing these findings, the curriculum and protocol was revised. The *pilot study* in two practices successfully implemented the protocol as follows: all seven providers were trained in two 45 min didactic education and skills session; incorporation of OH questions into practices EMR; recruited 86 child-parent dyads (95% participation) at the WCV; providers delivered the OH intervention to parent/caregivers in <2 min and 90% completed EMR documentation of OH questions. These findings were instrumental in finalizing the main PACT trial in 18 practices. The RE-AIM framework is used in the main trial to collect effectiveness and implementation measures at baseline and follow-up visits. **Conclusions**: The formative and pilot findings were instrumental in refining the OH intervention and protocol which has resulted in successful implementation of the main trial. **Trial Registration**: Clinical trials.gov, Registered 9 November 2017, NCT03385629.

## 1. Background

Dental caries (tooth decay, cavities) has a broad impact on overall well-being of a child, and can cause significant pain and progress to infection, abscess, cellulitis, and even death [1]. Pain associated with caries, impairs ability to chew, interference with sleep, missing school, and impaired academic performance [2]. Systematic reviews and meta-analysis indicates that obese, overweight, or underweight children to have significant increased risk for early childhood caries [3,4,5]. Although caries experience and untreated caries among 2–8 year old children have decreased nationally, poor and minority children still have a higher burden of disease compared to their more affluent counterparts [6]. Our community-wide studies in 5- to 6-year-old school children indicate an untreated primary caries rate of 42% [7], much higher than the national average of 14% [8]. The 2016 national estimates indicate that 46% of Medicaid enrolled 2- to 18-year-old children had received preventive dental visits, while in Ohio it was 35% [9]. For 13% of Medicaid-enrolled young children, their first dental visit was for emergency care [10]. About 84% of Medicaid-enrolled 1–2 year olds and 63% of 3–6 year olds received a well-child primary care visit. However, in contrast, only 9% of Medicaid-enrolled 1–2 year olds and 38% of 3–5 year olds received a preventive dental visit despite anticipatory guidance for dental visits starting from age 1 [11,12,13].

Pediatric Providers Against Cavities in Children’s Teeth (PACT) is one of four consortium clinical trials funded by the National Institutes of Dental and Craniofacial Research aimed at reducing childhood oral health disparities. The PACT study is testing theory-based multi-level interventions at the practice and provider (pediatrician, nurse practitioners) levels to deliver core oral health facts to parent/caregivers for enhancing self-management strategies and seeking dental care for their child’s oral health issues, and document such activities in electronic medical records (EMR) for enhancing patient care quality and sustainability. A recent systematic review by the U.S. Preventive Services Task Force (USPSTF) to update its recommendation for medical primary care clinicians concluded that there is a lack of evidence on effectiveness of parent/caregiver educational interventions and primary care referral to dentists [14]. 

Surveys of pediatricians’ and family physicians’ attitudes toward and engagement in oral health activities (i.e., screenings, risk assessments, counseling, referral to dentists) reveal that they support these activities but also lack confidence performing them [15,16]. Interviews with parent/caregivers [17] have found that they value their pediatricians as an important source of oral health information. Pediatricians’ prompting can help facilitate children’s first dental visit [17], while their contradictory messages/inadequate information can lead to caregivers’ confusion [18]. The few retrospective cohort studies include: One that examined the effectiveness of non-dental primary care providers delivering preventive oral health services (POHS: such as screening exams, dentist referral, and fluoride varnish) to young Medicaid-enrolled children found that it resulted in lower decayed missing filled teeth (DMFT) in kindergarteners who received POHS compared to those who did not [19]. However, a recent study found contradictory results that caries-related expenditures among children < 6 years old (using Alabama Medicaid claims data) were not different between those receiving early preventive care from a primary care provider versus not [20].

The literature is clear that most low-income parent/caregivers see dental diseases as acute (i.e., to be responded to only when there is pain or visible decay) [21]. One approach shown to be useful in changing this reasoning process and for self-management of chronic medical conditions is the Common-Sense Model of Self-Regulation (CSM) [22,23]. The CSM is a psychological approach where individuals create a mental representation (or perception) of their illness based on the abstract and concrete sources of information available to them. Recently, this framework has been utilized in behavioral interventions to improve the cognitive and emotional representations of parent/caregivers of children with caries [24]. Historically, oral health education providing factual knowledge to parent/caregivers with the intent of improving specific oral health behaviors has largely been ineffective [25,26]. Therefore, in the PACT trial, the providers will be trained to deliver key CSM theory-based oral health facts to parent/caregivers at well-child visits (WCV).

Since dental caries has physical and psychosocial impacts on a child’s well-being, it is appropriate to explore multidisciplinary and inter-professional strategies to increase dental utilization. We describe the formative and pilot work objectives that were accomplished to make changes to the main hybrid I trial. These objectives were to: (1) Conduct formative work (focus groups/key informant interviews) among primary care providers, practices, and parent/caregivers to assess the acceptability/barriers for oral health activities at WCVs, and gain input regarding the experimental practice and provider level intervention; (2) Pilot test the experimental intervention and implement the protocol in two primary care practices with providers and parent/caregivers of 3–6 year old Medicaid-enrolled children to determine feasibility for the larger main trial.

## 2. Methods

The PACT trial is using an effectiveness-implementation hybrid I design [27]. From an effectiveness standpoint, the reinforcement of CSM-based OH facts from the pediatrician along with a multi-component parent-level intervention represent novel behavioral enhancements to seek dental care for children which has never been attempted. The cluster randomized trial also employs a comprehensive implementation strategy to support the use of interventions in the primary care setting.

This section is organized into the two phases—Phase 1: Formative Work and Phase 2: Pilot Study. The Institutional Review Board of University Hospitals Cleveland Medical Center approved the study protocol. Written consent was obtained from all formative work and feasibility study provider and parent/caregiver participants. The study satisfies the COREQ and CONSORT requirements and has been included. 

### 2.1. Phase I: Formative Work (Focus Groups and Key Informant Interviews)

#### 2.1.1. Design

Six focus groups were conducted using community leaders/members, pediatric primary care providers (pediatricians or nurse practitioners), and parent/caregivers from two primary care pediatric practices. Inclusion criteria for practices were: (1) use of an electronic medical record (EMR), and (2) ≥20% of pediatric patients covered by Medicaid. Medicaid is a United States Federal and State program that provides free or low-cost health coverage for eligible low-income individuals. A purposive sampling of two practices from a group of thirty two pediatric Medicaid accountable care organization (ACO) practices were selected based on: one having 20–40% and the other > 40% of their patients enrolled in Medicaid. The inclusion criteria for community focus groups was that participants hold a leadership role in a community or neighborhood organization. Providers involved in the focus groups were pediatricians or nurse practitioners with a minimum of two patient-care days per week from the two practices. Caregiver participants from the two practices were required to be: (1) the legal guardian of a Medicaid-enrolled child who attended WCVs at two participating practices, (2) aged ≥18 years, and (3) English-speaking. Key informant interviews were conducted with practice leadership (i.e., medical director and clinical/office manager) from the two practices who were chosen because of their knowledge and experience, to serve as an “expert” on the practice. In all 24 community leaders, 7 providers, 12 parent/caregivers, and 4 practice leaders were selected to participate. All focus groups and key informant interviews (≈60 to 90 min) were conducted between September 2015 and July 2016. Written consent was obtained from all participants. 

Based on focus group methodology [28,29], a member of our project staff—an experienced moderator (MBS) trained in qualitative interviewing—used a semi-structured interview guide and open ended questions to gather a large number of opinions and engaged in a collective brainstorming of ideas and solutions. All focus groups were audio recorded, and two other project staff observed and took notes. Discussions were held at a community venue or the participating primary care practice. A semi-structured interview guide was developed for each group of participants: community member, provider, or parent/caregiver. Interview questions were focused on several key aspects of feasibility including acceptability, demand, implementation, practicality, and adaptation [30]. 

The interview guide for *community members* focused on the acceptability and demand for an oral health intervention, barriers for dental care, and available resources in the community. The *provider* guide addressed the practicality and implementation of an intervention, such as the content and length of the oral health curriculum, overall logistics of OH integration into WCVs, and documentation and implementation of oral health protocols. The *parent/caregiver* guide concentrated on the acceptability and practicality of primary care provider communication of OH facts (i.e., what they would like to know), OH activities being performed at WCVs (i.e., dental screening by hygienist), recruitment and retention strategies (i.e., participation motivators, contact methods, study incentives), respondent burden in questionnaire completion (paper vs. electronic, time length), and barriers or resources needed for dental access.

Key informant interviews were conducted with the *practice-level leaders* (i.e., medical director and clinical/office manager) to gain input on the implementation of a dental study in a pediatric primary care setting. Key informants were interviewed by the same moderator (MBS) in a private room at each of the primary care practice. All interviews were audio recorded. 

#### 2.1.2. Analysis

Focus group and interview discussions were transcribed, coded, and analyzed using Atlas.ti (version 7, Scientific Software Development GmbH, Berlin, Germany). The transcriptions were verified by two different members of the project staff. Then, the same two individuals (i.e., coders) independently read and coded the transcripts using methods of both theory- and data-driven coding [31]. With theory-driven coding, based on grounded theory methodology, codes were assigned to concepts or questions from the interview guides. Data-driven, or open, coding followed an inductive approach, and codes were generated based on emerging themes [31]. Each coder devised their own coding scheme before coming together and finalizing the mutually agreed upon codebook. 

Based on the formative work findings, the provider curriculum, protocol, logistics, and data collection methods were further refined prior to pilot testing.

### 2.2. Phase II: Pilot Study

#### 2.2.1. Study Design and Practice Sites

The two practices utilized for the focus groups participated in the pilot study. In phase II, the feasibility of the experimental intervention was tested in terms of intervention training, data collection, and measures, fidelity monitoring, and study logistics at WCVs. While the same providers participated in both the focus groups and pilot study, different parent/caregiver participants were chosen for each. The pilot study was conducted between August 2016 and April 2017.

#### 2.2.2. Practice Intervention

Based on the formative work, the OH questions were finalized for incorporation into each practice’s EMR. The two study practices were given the option of whether or not to group the OH questions together; and automatic generation of the prescription and list of dentists depended on the capabilities of each practice’s EMR.

#### 2.2.3. Pediatric Primary Care Providers

*Recruitment*: Provider selection criteria was a minimum of two patient-care days per week. All providers in the two practices were invited to participate in the study. Project staff obtained written consent from providers for the pilot study. The providers were given one continuing medical education (CME) credit for the didactic training.

*Provider and Practice Interventions and Training*: Prior to parent/caregiver pilot study recruitment, providers received training in two 45-min sessions: CSM-based oral health didactic presentation and skills training with standardized patients. The didactic presentation was adapted from two existing training modules [32,33] to include the following CSM-based OH constructs: caries etiology and risk factors (*identity and cause*), impact of caries on health and well-being (*consequences*), importance of primary teeth (*timeline*), and preventive care such as oral hygiene, cariogenic diet, and dental visits (*controllability*). Training materials (training manual, flip chart, and pocket card) were developed and then used at the didactic presentation and the skills training. The pocket card was used during the skills training to maintain the fidelity of provider delivery of the six CSM-based OH facts (i.e., take home messages for parent/caregivers) to standardized patients. 

Additionally, for the practice level intervention, the two practices incorporated four OH questions in their EMR for systematic documentation by the provider.

*Data Collection and Measures*: Providers completed a pre- and post-test questionnaire before and after the training to evaluate providers’ OH knowledge. In addition, providers documented the four finalized OH questions (yes or no responses) in EMR: (1) examined child’s teeth for white or brown spots, (2) asked whether child had a dental visit in the past 12 months, (3) communicated core OH facts, and (4) gave caregiver a prescription to take their child to the dentist along with a list of Medicaid-accepting dentists in the area.

*Fidelity Monitoring*: To assess the feasibility of fidelity monitoring for the main trial, the following methods were tested: observations by study staff, provider audits of OH documentation in EMR, and parent/caregiver exit surveys regarding their OH encounter with providers. Each provider was observed by study staff using a standardized skills checklist (provider relationship skills such as confidence, honesty, enthusiasm, open to parent/caregiver perspective and questions; communication of the six core OH facts to the parent/caregiver; other communication elements such as motivating and encouraging parent/caregiver; and provision of prescription and list of dentists) at the WCV. Providers were given immediate feedback from these observations. Additionally, the four OH questions added to the EMR were abstracted and analyzed to see if the providers were documenting their OH encounters with patients; the results were presented to the providers in a scorecard. Similarly, the parent/caregiver responses to the exit questionnaire about their OH encounter with providers were analyzed and presented in a scorecard.

#### 2.2.4. Parent/Caregiver Child Dyads 

*Recruitment*: Parents/caregivers of 3- to 6-year-old Medicaid-enrolled children were approached for participation during WCVs at the two practices from September 2016 to April 2017. Project staff obtained written consent from eligible participants. Participants received a $25 cash incentive for their time to participate in the study.

*Data Collection and Measures*: Study staff collected responses to three surveys, and sent a follow-up survey to be completed and returned within a month. All children were examined by the dental hygienist at the WCV. Pilot study data collected for refinement of the main trial was: (1) time added to the WCV due to the addition of OH activities; (2) % parents willing to participate; (3) % children with cavities; (4) % follow-up surveys returned; (5) % parents who took their child to the dentist or scheduled an appointment within eight weeks of the WCV. 

## 3. Results

### 3.1. Phase 1: Formative Work (Focus Groups/Key Informant Interviews)

#### 3.1.1. Recruitment

A total of 26 participated in the focus group sessions. Twenty-four community members were selected to participate in the community member focus groups and 15 attended (eight in the first focus group and seven in the second). All seven pediatric primary care providers (four pediatricians and three nurse practitioners, 100% female) participated in two consecutive focus groups. Twelve parent/caregivers were scheduled to participate in the parent/caregiver focus groups and five attended (80% female and 20% male) each of the two focus groups. All four key informants (i.e., the medical director and the office/clinical manager at each of the two practices) approached agreed to participate (100% female). 

#### 3.1.2. Acceptability

Community member and parent/caregiver suggestions that OH be discussed at the WCV specifically mentioned that pediatricians should provide information or recommendations for dental visits (i.e., age of first visit, frequency of visits), age-appropriate self-care strategies (i.e., tooth brushing, oral health products, nutrition), and a list of area dentists who accept young children and Medicaid. Community members also highlighted the barriers to dental care (finding Medicaid-accepting dentists) for Medicaid enrolled children but confirmed that parents were more likely to take their child to the pediatrician’s office annually. Other concerns, such as barriers to research participation, are presented in Table 1.

#### 3.1.3. Demand

Feedback from focus groups indicated a demand for OH training and improvement in OH knowledge and resources, such as a list of dentists, to better inform parent/caregivers. For example, primary care providers admitted they lacked knowledge regarding the chronicity of dental caries or its timeline, specifically the progression of the disease, and the impact of cavities in primary teeth on newly erupting permanent teeth. Another area of confusion was the age at which young children should start seeing the dentist. Focus group participants and informants were in agreement about what caregivers should know about children’s oral health: causes of dental caries, what healthy vs. unhealthy teeth look like, and self-care strategies to help caregivers take care of their child’s teeth. Primary care providers, in particular, identified the consequences of dental caries as important for caregivers to know. 

#### 3.1.4. Implementation and Practicality

For providers, training materials (training manual, flip chart, and pocket card) were developed for the skills training and to aid in implementation of OH activities during the WCV. A pocket card containing scripts was created (i.e., take home messages and opportunities to introduce OH in the WCV) to ensure that curricula are delivered efficiently. In terms of logistics, practical concerns such as respondent burden, time, and physical space were considered in the implementation of OH activities during WCVs. Project and practice staff acknowledged the need for relationship building and clear communication to ensure that study participation did not add or take away from provider-patient time. Issues like space and logistical flow were determined to be practice-specific.

#### 3.1.5. Adaptation

Primary care providers suggested that, for easy adaptation, the EMR documentation of OH activities be mindful of the time constraints of a busy provider. They were willing to document oral health questions if the EMR documentation was made easier by limiting the number of questions. As a result of this, four key oral health questions were incorporated into the two practices’ EMR. 

### 3.2. Phase II: Pilot Study

#### 3.2.1. Study Participants

At the two pediatric primary care practices, all seven providers consented to participate in the pilot study. From those providers’ patients, a total of 91 parent/caregivers were approached and 86 consented to participate (95%) in the pilot study. 

#### 3.2.2. Provider Intervention Training

The provider didactic and skills training sessions each took 45 min on average. This included administration and collection of study-related consent and questionnaires. The short duration of these training sessions provided flexibility for providers to attend during their lunch breaks when no patient appointments were scheduled. All seven enrolled providers completed pre- and post-tests. The didactic training resulted in an overall 11% improvement in providers’ oral health knowledge based on the pre-test (86%; *M* = 14.57, *SD* = 1.72) and post-test (97%; *M* = 16.60, *SD* = 0.89) scores. 

#### 3.2.3. Practice Intervention

Based on the formative findings, four core OH questions were incorporated into each practice’s EMR. Providers’ answers to the four questions indicated (yes or no) whether they: (1) examined child’s teeth for white or brown spots, (2) asked if child saw dentist in past 12 months, (3) communicated oral health facts, and (4) gave prescription and list of dentists. Documentation for all providers in these two practices was 90% complete for all four OH questions.

#### 3.2.4. Intervention Fidelity

Project staff observation of OH encounters indicated that 92% of the core OH facts were being communicated to the parent at the WCVs—evidence that providers were implementing the intervention with fidelity. Parent/caregivers also confirmed this through the exit survey by reporting on their provider’s ability to give useful oral health information during the WCV. Nearly 92% (*n* = 79) of parent/caregivers felt that their provider communicated core OH facts “very well” or “well”, and 8% (*n* = 7) “adequately”. 

#### 3.2.5. Results from Parent–Child Pilot Data

*Study outcomes*: A total of 51 out of 86 Medicaid-enrolled children aged 3 to 6 years old had dental caries (59%). About 59% of the parent/caregivers reported that they either took their child to the dentist or made a dental appointment within two months of the WCV. 

*Questionnaire completion*: All 86 enrolled caregivers completed 100% of the in-person surveys while at the WCV. The return rate for the follow-up questionnaires collected via mail or text message was approximately 80%. Parent/caregivers were asked for their opinions on the difficulty and length of the study. The majority—68.6% (*n* = 58)—reported that the length of the surveys was “about right”, 31.4% (*n* = 27) reported that the length of the surveys was long, and 1.2% (*n* = 1) felt it was “too short”. Over 87% (*n* = 75) agreed that the surveys were “not difficult” and only 13% (*n* = 11) reported the surveys were “moderately difficult”. 

*Time length for oral health activities*: The incorporation of the OH components into the WCV added less than 5 min, including the hygienist exam and the provider’s delivery of OH facts. Consent and questionnaire completion added about 15 min without disruption to the providers’ schedule as they were completed before or after the provider saw the child.

### 3.3. Main Hybrid I Trial

**Figure 1:**
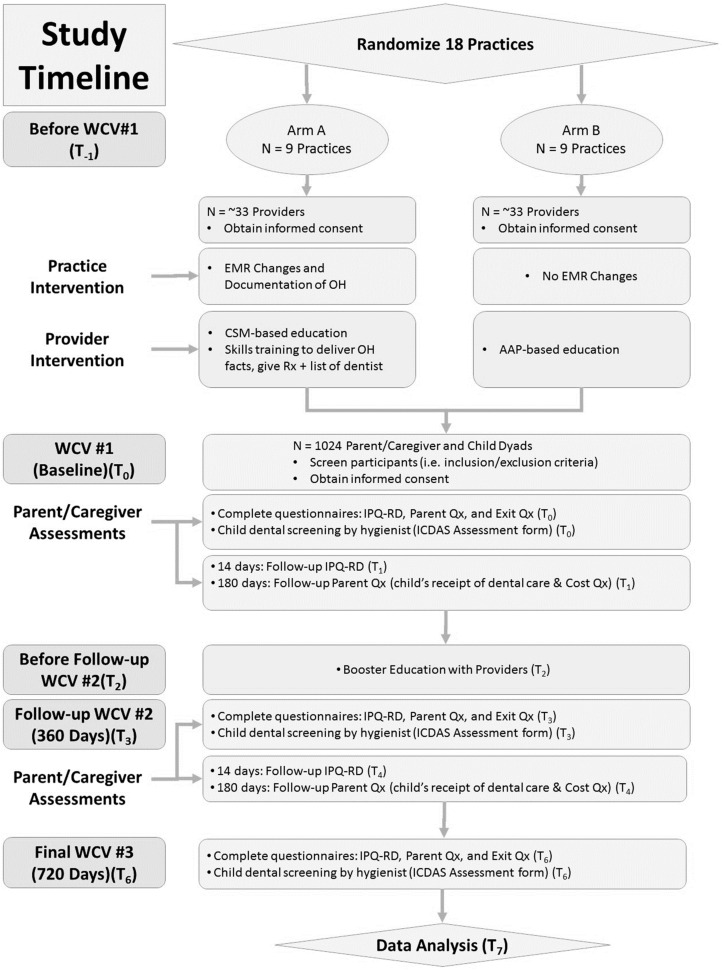
Study design of the main trial.

Based on our formative and pilot findings, the study design was finalized to be a two-arm parallel design (Figure 1) and transition to the main trial occurred in 2017. 

Sample size and power for the main trial were calculated with dental attendance being the primary outcome. The use of a two-sided 0.05 alpha level *z*-test (with pooled variance) for a difference in proportions was used. The effect size was calculated as 16% difference between Arm A with 46% (based on national estimates) [34] and for Arm B as 30% (based on this pilot study data for routine dental visits in past year). Making conservative allowances for an intra-cluster (within-practice) correlation (ICC: in the binary dental attendance outcome) of 0.04 and a 25% drop-out rate, a sample size of 512 participants per arm (total *n*= 1024) provides an estimated 80% power to detect the above difference in proportions. For the purposes of this study, a sample size of 512/arm (*n* = 1024) will be recruited.

Subsequently, 18 practices and 1024 child-parent dyads have been recruited and the trial is currently in progress and will be completed in Fall of 2023.

#### Conceptual Model for the Main Trial

**Figure 2:**
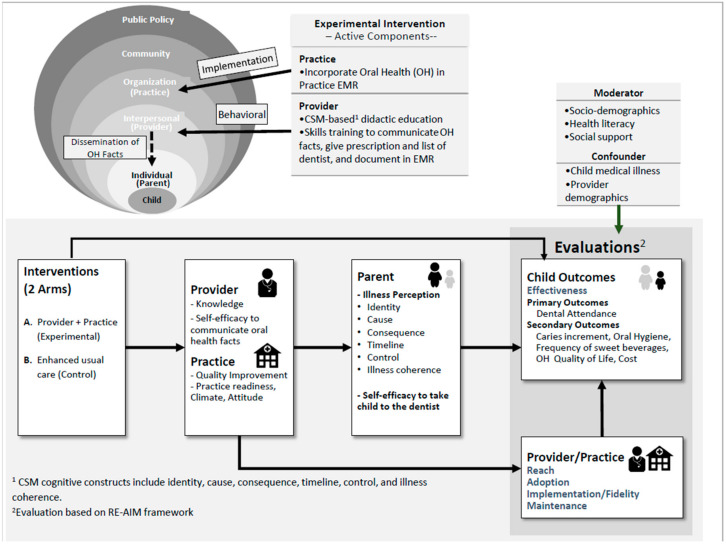
Conceptual model of the main trial.

The focus of the cluster randomized trial is on addressing factors (determinants) at the three socio-ecological levels of the child’s environment: parent/caregiver, provider (pediatrician), practice/organization levels (Figure 2). Figure 2 illustrates the pathway through which the interventions are intended to result in improved child outcomes. Thus, the pediatric provider’s communication of OH facts + written prescription and resources are intended to change parental caries illness perception (from disjointed inaccurate → chronic organized understanding) and self-efficacy to seek dental care for child. On a practice level, the integration of oral health and systematic documentation in EMR supports uniform data collection and enhances continuous quality improvement to facilitate follow-up with the parent at the next WCV. Study evaluations will utilize the RE-AIM framework [35,36]. Internal validity is assessed by effectiveness and implementation/fidelity; external validity is assessed by reach, adoption, and maintenance. The child primary and secondary effectiveness outcomes will be mediated by changes in parent’s illness perception and self-efficacy (Figure 2). Other external validity outcomes as a result of provider and practice level interventions via mediators will also influence the child outcomes. Moderating variables for the model included: parent’s socio-demographics [37], health literacy [38], social support [39,40], and child medical illness [41,42].

## 4. Discussion

Results from the preliminary work indicate that a theory-driven oral health intervention is feasible for practices, providers, and parent/caregivers. Considering dental caries in children has broad impact on systemic and psychosocial health, we developed and tested a CSM-theory based oral health curriculum, including skills training, for pediatric providers (to be delivered in two 45-min sessions which busy providers could attend during their lunch break); development of core oral health facts which providers can deliver to parent/caregivers during WCVs in <2 min; and EMR documentation of oral health activities for quality improvement and follow-through with caregiver at subsequent WCVs. Additionally, provider OH knowledge increased because of the didactic and skills training, thus indicating the validity of our theory-based curriculum. The pilot study findings indicated that the intervention can be implemented according to the practice constraints of limited provider–patient encounter time and physical space. Chiefly, parent/caregivers triangulated this evidence by confirming that the study activities were not challenging and manageable in terms of length and time required to complete. Parent/caregivers also reported that providers were able to effectively communicate core OH information during the WCV. 

An effectiveness-implementation hybrid I design [27] was used in this cluster randomized trial for the following reasons: the lack of evidence-based oral health (OH) behavioral interventions that can be implemented at this time; preliminary evidence from our seminal work for the innovative CSM-based interventions that would allow testing in a new setting and population; strong “implementation momentum” (i.e., recommendations from American Academy of Pediatrics and American Academy of Pediatric Dentistry) towards routine adoption of OH assessments within the primary care setting for children up to 6 years old; and access to primary care practices to test the interventions that would support generalizability. This hybrid design has been successfully employed in other medical research [43,44] but has not been used in dental research.

### 4.1. Provider Intervention 

Specifically, the formative research findings indicated the need for an OH curriculum to change pediatric primary care providers’ perception of the chronicity of dental caries. Providers acknowledged that they did not have a clear understanding of: (1) carious primary teeth (baby teeth) being a risk factor for newly erupting permanent teeth, or (2) the age at which children should receive their first dental visit. Insufficient oral health training and mixed messages regarding when a child should first see the dentist (i.e., incongruence between recommendations from professional organizations and dentists unwilling to accept Medicaid-enrolled preschool aged children) both contributed to providers’ misconceptions. Parent/caregiver misperception regarding baby teeth [24] provides a perfect opportunity for pediatric primary care providers to communicate core oral health information at WCVs. Hence, the CSM theory was used to develop this curriculum [24]. The CSM has been used previously in other clinical trials to improve treatment adherence of patients with chronic illnesses such as myocardial infarction [45], diabetes [46], and chronic back pain [47]. Results from our pilot study also suggest that providers can successfully impart these theory-based oral health facts to parents at a WCV.

The core elements of the provider OH curriculum correspond to CSM constructs (identity, cause, consequences, timeline, and control) to change the perception of dental caries to a chronic organized model [24]. Provider and parent/caregiver perspectives were combined to streamline the didactic education and skills training while maintaining the theory-based curriculum. More emphasis was placed on aspects of the curriculum with which providers were less familiar (i.e., identity, cause, and timeline of dental caries); less emphasis was placed on the consequences and controllability of dental caries about which providers were more knowledgeable. To address providers’ concerns about length and scheduling, the oral health curriculum was split into two 45-min sessions: first, the didactic education and second, the skills training. To maintain the fidelity and consistency of curriculum delivery for the 18 practices in the main trial, the study team has subsequently developed a narrated presentation and a video simulation of oral health activities at the WCV that is now part of the didactic session.

To ensure that providers deliver the intervention (i.e., communicate core oral health facts to caregivers) with fidelity, providers needed resources, such as a training manual, informational flip chart, and pocket card (with the core oral health facts) as well as a list of Medicaid-accepting dentists in the area. The list of dentists is an essential resource because pediatric primary care providers in prior studies have been hesitant to integrate oral health into their practices because there were few dentists to whom they could refer their Medicaid-enrolled patients [48,49]. For the pilot study and the main trial, we developed practice-specific lists of Medicaid-accepting dentists in the immediate surrounding areas to facilitate the referral process.

### 4.2. Practice Intervention 

To facilitate adoption of EMR documentation, the number of oral health questions was restricted to 4 yes/no questions (i.e., examined teeth for white or brown spots, asked if child saw dentist in past 12 months, communicated oral health facts, gave prescriptions, and list of dentists). The two pilot practices were given the option of whether or not to group the oral health questions together; automatic generation of the prescription and list of dentists depended on the capabilities of each practice’s EMR. Quality improvement (QI) studies have found that requiring providers to document the delivery of oral health services in EMR formalizes the behavior and increases the likelihood that it is performed [50,51]. Our pilot study showed that the four oral health questions can be easily incorporated into any practice’s EMR and documented by providers.

#### 4.2.1. Recruitment and Retention Strategies

Input from all four groups on successful recruitment and retention strategies were supported by previous research—most importantly, project branding [52] and highlighting the social relevance of research to motivate participation [53,54]. Suggestions for project branding included the creation of a study logo to be put on study materials and giving incentives to child participants (i.e., stickers, tooth brushing chart, pouch for tooth brushing, or school supplies). One strategy repeatedly proposed in discussions was to emphasize to caregivers the importance of children’s oral health and the resources they would be given (i.e., list of area dentists who accept Medicaid, family tooth care kit with toothbrushes and toothpaste). Prior studies have demonstrated that African Americans, in particular, are motivated to participate in health research that has relevance (or value) to or can help them, their family, their community, or even minority communities in general [53,54]. Employing these strategies in our pilot study, we were successful in recruiting 95% of the parent/caregivers approached for participation. Similar strategies were followed in our main trial that resulted in recruiting the required sample of 1024.

#### 4.2.2. Flow of Intervention Delivery

Providers and practice leadership offered input on how oral health activities could be incorporated into the WCV with less interruption of their practice routine. For recruitment purposes, a script for practice staff was developed to use during reminder calls with eligible patients. In our pilot study, medical assistants helped by identifying eligible patients/caregivers and approaching them first (i.e., demonstrating providers’ support of the study) before consent was obtained by project staff. This was an effective strategy in reducing interruption to the practice routine (and improving recruitment) as reported previously [55]. 

#### 4.2.3. Other Implementation Considerations

Discussions during the formative research highlighted the importance of considering participant preferences [53] as follows: caregivers should be asked how they would prefer to be contacted by study staff and/or receive study information; and caregivers should be given the option of completing study visit and follow-up questionnaires on paper or electronically (i.e., tablet, text with link). In the pilot study, participants were asked for their preference with the goal of reducing respondent burden as well as to improve retention to remain in the study longer term. These strategies were helpful in achieving an 80% questionnaire return rate and in contacting the parents after the WCV to inquire whether their child received dental care.

#### 4.2.4. Strengths/Limitations/Challenges

The strengths of the study are that the formative and pilot work were necessary and instrumental to refine sample size, logistics of recruitment, and plan for the larger community wide trial. It would have been impossible to implement the trial without such preliminary groundwork and implement successfully. A methodological limitation was that only two practices were selected for the pilot study and the lessons learned in these practices underestimated the recruitment timeline for the larger trial. Providers also indicated challenges in referring young Medicaid enrolled children to a dentist. The most notable issue was that few dentists accept Medicaid insurance. The second was confusion surrounding messaging by dentists that young children do not need to see a dentist until three years old rather than 1 year old, as recommended by the AAPD. To address the shortage of Medicaid-accepting dentists, project staff gave each of the practices an updated list of dentists after calling dental offices in the surrounding areas (of the practice) and confirming their acceptance of Medicaid insurance. In terms of inconsistent messaging by dentists regarding the recommended age for a child’s first dental visit, the project staff acknowledged that this was an area for future work. In our pilot study, the high participation rates among parent/caregivers may have been motivated by cash incentives. However, incentivizing is necessary to compensate individuals for their time added to their child’s WCV. 

## 5. Conclusions

Considering the profound implications of the oral-systemic link in children, this study offers an expanded perspective on the importance of formative research and pilot study for the refinement of a larger hybrid I trial integrating oral health activities into several pediatric primary care practices. 

## Figures and Tables

**Table 1:** Summary of formative work incorporated into the main trial.

Feasibility Objective	Recommendations Based on Focus Groups/Key Informant Interviews	Changes Made to the Feasibility/Main Study
**Acceptability**	Community members and parent/caregivers suggested that it is important to discuss oral health at the WCV Barriers to caregivers participating in research included: Respondent burden Incentives	Offer caregivers options to complete paper or electronic surveys Use tablet computers for data collection Cash incentive for each WCV and additional gift card incentives for completing follow-up surveys Revised instructions and scales based on analysis of missing responses and skip patterns to make surveys more succinct and less confusing
**Demand**	What primary care providers informed that they do not know about: Risk factors for dental caries Importance of baby teeth Chronic nature of dental caries Identification of abnormalities in teeth and mouth Age at 1st dental visit Provide resources such as list of Medicaid-accepting dentists	Integrated CSM theory-based information into the didactic and skills curriculum. Area Medicaid-accepting list of dentists created for each practice. Oral health materials included the age at which the 1st dental visit should start.
**Implementation**	Important OH information to be communicated by providers at WCV: Causes of dental caries Importance of baby teeth Consequences of dental caries Age-appropriate self-care strategies Recommendations for preventive dental visits (age at 1st and frequency) Flow of activities during WCV	Six key OH facts based on the CSM were developed to be easily communicated by the provider during the WCV. Pocket cards, training manual, flip charts created. Didactic training session to be delivered as a narrated slide presentation for content and time consistency. Also includes a video documentary of the walk-thru of oral health activities at the WCV. Fidelity monitoring plan updated and revised.
**Practicality**	Recruitment script for practice staff to use Method of contact with parent based on their preference (via mail, phone, voicemail, text, email) Incentives to be given to children as well as caregivers	Created scripts for office staff to talk about the dental study. Medical assistant approaches eligible caregivers first before consent is explained and obtained by study staff. Offer caregivers options to complete paper or electronic surveys (e.g., tablet, text with link to online survey)
**Adaptation**	OH documentation in EMR should be: Reduced to a few questions grouped together in EMR	Four questions (with yes/no responses) integrated into each practice EMR

## Data Availability

The datasets used and/or analyzed during the current study are available from the corresponding author on reasonable request.

## Abbreviations

AAP: American Academy of Pediatrics, AAPD: American Academy of Pediatric Dentistry, ADA: American Dental Association, CSM: Common-Sense Model of Self-Regulation, EMR: Electronic Medical Record, OH: Oral Health, PACT: Pediatric Providers Against Cavities in Children’s Teeth, QI: Quality Improvement, RCC: Rainbow Care Connection, USPSTF: U.S. Preventive Services Task Force, WCV: Well-Child Visit.
